# Estimated excess mortality figures in the year 2020-2021 in casentino municipalities (Italy)

**DOI:** 10.1093/eurpub/ckac131.068

**Published:** 2022-10-25

**Authors:** M Gennari, M Fratini, N Nante, N Vigiani

**Affiliations:** 1 Post Graduate School of Public Health, University of Siena, Siena, Italy; 2 Department of Molecular and Developmental Medicine, University of Siena, Siena, Italy; 3 UOC Food and Nutrition Hygiene-East Area, Azienda USL Toscana Sud-Est, Arezzo, Italy

## Abstract

**Background:**

In 2020, the total deaths from all causes were the highest ever recorded in Italy since World War II (15.6% excess), with peaks during Sars-Cov2 waves and reductions in periods of national lockdown. On the contrary, in 2021, the total number of deaths from all causes reduced compared to the previous year. Analysing local mortality data, our study aims to assess whether, in the Casentino Valley (Arezzo, Tuscany), an excess of deaths from all causes occurred between January 2020 and December 2021 compared to the five years 2015-2019.

**Methods:**

We used the official mortality data from the Italian Institute of Statistics (ISTAT), and that are published in the Table of deaths by the municipality on March 2, 2022. From this database, we extrapolated all deaths from all causes between January 1, 2020, and December 31, 2021, in the 10 municipalities of Casentino. The data collected were processed using Microsoft Excel 2016 software. We then distinguished by total per month, gender, and age group and then compared these data with the previous five years’ average by calculating the percentage change.

**Results:**

Overall, both the years 2020 and 2021, it is shown an increase in the deaths percentages compared to the previous five-year period, respectively 5.66% and 8.07%. In particular, there is an excess of mortality in November and December 2020. The increase in mortality is more remarkable for males (13% in 2020 and 20% in 2021). The highest percentage increase was recorded in 2021 for the 75-84 age group (+15%) and in 2020 for males over 85 (+29%).

**Conclusions:**

The data analysed confirms the excess mortality in 2020-2021 compared to the average of the previous 5 years in Casentino. There are peaks in November and December 2020, corresponding with the second wave of Sars Cov2 infection. The results obtained establish the basis for subsequent analyses that will verify the correlation of mortality peaks with the incidence of Sars-Cov2 cases in the territory studied.

**Key messages:**

• Between January 1, 2020, and December 31, 2021 there was an excess of all-cause mortality in our area.

• This excess mortality appears to be related to peaks in Sars-Cov2 infection.

